# Our Journal as an Advertising Medium

**Published:** 1867-02

**Authors:** 


### OUR JOURNAL AS AN ADVERTISING MEDIUM.

We take this method of informing the public that our circulation in Georgia, and, indeed, in all of the Southern States, is perhaps equal to that of any other Southern journal, and we respectfully solicit a share of the patronage appropriate to such a medium.

